# Editorial: Natural bioactives used as additives in food applications

**DOI:** 10.3389/fnut.2022.1063942

**Published:** 2022-11-08

**Authors:** Carla Pereira, Lillian Barros, Celestino Santos-Buelga, Miguel A. Prieto

**Affiliations:** ^1^Centro de Investigação de Montanha, Instituto Politécnico de Bragança, Bragança, Portugal; ^2^Laboratório Associado para a Sustentabilidade e Tecnologia em Regiões de Montanha, Instituto Politécnico de Bragança, Bragança, Portugal; ^3^Grupo de Investigación de Polifenoles, Universidad de Salamanca, Salamanca, Spain; ^4^Nutrition and Bromatology Group, Department of Analytical Chemistry and Food Science, Faculty of Science, Universidade de Vigo, Ourense, Spain

**Keywords:** bioactives, additives, food industry, green extraction, sustainability

Since ancient times, different natural products have been used to enhance the attributes of foodstuffs, including sensory characteristics, nutritional value and also extend the shelf-life. More recently, these natural products have been also reported to exert beneficial biological properties in the health of consumers. For these reasons, numerous recent studies have been focusing on the valorization of bio-based extracts and compounds for their application in different industries, especially in the food sector. Moreover, considering that food-derived products not intended for consumption can be rich in bioactive compounds, many efforts have been made to employ by-products from food industry to extract such compounds and contribute to circular economy approaches.

Nowadays, additives play a fundamental role in food industry and their use has increased worldwide, most of them being of artificial origin. To overcome safety issues and prioritize natural sources, bio-based additives have attracted the attention of the scientific community and industry, aiming at meeting consumers' expectations. In this context, natural extracts and compounds have been intensively explored in recent years as new food additives, paying particular attention to the sustainability of the extraction process for further application in industry. Innovative technologies are necessary to reduce the time, costs, and losses of the processes, and to increase the extraction yield and extracts quality. On the other hand, the search for promising safe and green solvents is of crucial importance.

This Research Topic aimed to gather the most recent advances in natural ingredients and sustainable processes with high potential of application in food industry, publishing new reports in this field, as well as updating current knowledge. In this sense, the articles collected deal with the evaluation of natural extracts and compounds as possible food additives, most of them obtained from food industry residues, such as coffee by-products (Rebollo-Hernanz et al.), medicinal and aromatic plants residues (Dina et al.) or chicken cartilage (Zhang et al.). Some studies have employed innovative approaches for the extraction of compounds, such as ultrasonic-assisted extraction (Dina et al.), hot-pressure assisted extraction (Zhang et al.), or enzymatic extraction (Yu et al.). Besides extraction, another key point is to assess the biological and functional properties of the obtained natural extracts and molecules to confirm potential functional properties. Several studies have evaluated the antioxidant and anti-inflammatory activities and the modulatory effects of natural extracts *in vitro*, using spectrophotometric and cell-based assays (Dina et al.; Rebollo-Hernanz et al.). In other works, an in-depth evaluation of the action mechanisms related to the positive effects was carried out, including the activation of molecular signaling pathways and the modulation of gene expression (Rebollo-Hernanz et al.; Wang et al.). Additionally, some *in vivo* studies assessed antioxidant, anti-inflammatory and anti-osteoarthritis properties (Yu et al.; Zhang et al.). Thus, the reported findings could serve as a basis for future research of these matrixes as feed additives with diverse functional properties and high-added value. A research conducted in hens found that the inclusion of dregs of *Cardamine hupingshanensis* in the food improved health status and also the quality of eggs (Yu et al.). Another study presented new insights of natural products of vegetal and animal origin that have been employed as food additives to improve the quality of chicken eggs (Obianwuna et al.).

Concluding, this Research Topic compiles 6 peer-reviewed articles covering several relevant points involved in the valorization of bio-based extracts and compounds, including extraction, assessment of biological and functional properties and the mechanisms of action, the use of food industry by-products as sources of bioactive extracts and the evaluation of the potential use as food additives. Therefore, the original and review articles compiled provide some of the most up-to-date knowledge on the valorization of functional bio-based extracts and compounds and offer new insights for the progress of the food industry.

## Author contributions

All authors contributed equally to the work and have read and agreed to the published version of the editorial.

## Funding

The authors are grateful to the Foundation for Science and Technology (FCT; Portugal) for financial support through national funds FCT/MCTES (PIDDAC) to CIMO (UIDB/00690/2020 and UIDP/00690/2020) and SusTEC (LA/P/0007/2021), for LB and CP's contracts through individual and institutional scientific employment program contracts. The GIP-USAL was financially supported by the Spanish Government (Project PID2019-106167RB-I00) and Junta de Castilla y León (Project SA093P20). The research leading to these results was supported by MICINN supporting the Ramón y Cajal grant for MP (RYC-2017-22891) and by Xunta de Galicia for supporting the program EXCELENCIA-ED431F 2020/12.

## Conflict of interest

The authors declare that the research was conducted in the absence of any commercial or financial relationships that could be construed as a potential conflict of interest.

## Publisher's note

All claims expressed in this article are solely those of the authors and do not necessarily represent those of their affiliated organizations, or those of the publisher, the editors and the reviewers. Any product that may be evaluated in this article, or claim that may be made by its manufacturer, is not guaranteed or endorsed by the publisher.

